# Tumor necrosis factor alpha and adalimumab differentially regulate CD36 expression in human monocytes

**DOI:** 10.1186/ar2133

**Published:** 2007-03-02

**Authors:** Jean Frédéric Boyer, Patricia Balard, Hélène Authier, Bruno Faucon, José Bernad, Bernard Mazières, Jean-Luc Davignon, Alain Cantagrel, Bernard Pipy, Arnaud Constantin

**Affiliations:** 1 EA2405, Université Paul Sabatier, IFR31, BP84225, 31432 Toulouse CEDEX 4, France; 2 GRCB40, Université Paul Sabatier, IFR31, BP84225, 31432 Toulouse CEDEX 4, France; 3 Service de Rhumatologie, Centre Hospitalier Universitaire Rangueil, 1 avenue Jean Poulhès, 31059, Toulouse CEDEX 9, France; 4 INSERM, U563, IFR30, BP 3028, 31024 Toulouse CEDEX, France; 5 INSERM, U558, Faculté de Médecine, 37 allées Jules Guesde, 31073, Toulouse CEDEX 7, France

## Abstract

In chronic inflammatory diseases, such as rheumatoid arthritis, inflammation acts as an independent cardiovascular risk factor and the use of anti-inflammatory drugs, such as anti-tumor necrosis factor alpha (anti-TNFα), may decrease this risk. The phagocytosis of oxidized low density lipoproteins (LDLs) accumulated in the subendothelium by mononuclear cells influences atherosclerosis and depends on CD36 expression. We investigated the role of TNFα and adalimumab, a human anti-TNFα monoclonal antibody widely used in human pathology, in CD36 expression in human monocytes. Human monocytes were prepared by adherence from whole-blood buffy-coat fractions from healthy donors. CD36 expression was assessed by RT-PCR and flow cytometry, with various TNFα or adalimumab concentrations. Implication of peroxisome proliferator-activated receptor (PPAR)γ in the regulation of CD36 expression was assessed using specific inhibitor or gel shift assays. The impact of redox signaling was investigated using quantification of reactive oxygen species, antioxidant and a NADPH oxidase inhibitor. The F(ab')2 fragment of adalimumab was isolated and its effect was analyzed. TNFα inhibits both CD36 membrane expression and mRNA expression. This inhibition involves a reduction in PPARγ activation. In contrast, adalimumab increases both CD36 membrane expression and mRNA expression. This induction is independent of the Fc portion of adalimumab and involves redox signaling via NADPH oxidase activation. CD36 expression on human monocytes is inhibited by TNFα and independently increased by adalimumab. These data highlight that pro-inflammatory cytokines and their specific neutralization influence the expression of cellular receptors implicated in atherosclerosis. Further studies are needed to investigate the clinical implications of these results in accelerated atherosclerosis observed in rheumatoid arthritis.

## Introduction

In chronic inflammatory diseases, such as rheumatoid arthritis (RA), systemic inflammation appears as an independent risk factor, contributing to increased cardiovascular mortality [1]. This high cardiovascular mortality reveals the existence of accelerated atherosclerosis, the pathogenesis of which may be associated with multiple factors, such as dyslipidemia, deterioration of insulin sensitivity, hyperhomocysteinemia and endothelial dysfunction [2,3]. Control of systemic inflammation using conventional drugs, such as methotrexate, or biological therapies, such as anti-tumor necrosis factor alpha (anti-TNFα), provides a means of preventing high cardiovascular mortality among RA patients [4,5].

Of the various molecular agents of inflammatory response, proinflammatory cytokines, and TNFα in particular, play a major role in the development of atherosclerosis. TNFα promotes the expression of adhesion molecules, such as vascular cell adhesion molecule-1, E-selectin and intercellular adhesion molecule, necessary for the flow of leucocytes into the sub-endothelial tissue [6]. It also promotes production of other proinflammatory cytokines and chemokines, such as IL1, IL6 and IL8. Along with interferon-γ, TNFα plays an important role in atheroma plaque rupture by inducing overexpression of matrix metalloproteinases by macrophages, leading to degradation of the collagen matrix vital to plaque stability [7]. In apolipoprotein-E deficient mice, which provide a valid research model for atherosclerosis, inactivation of the gene encoding TNFα significantly reduces the size of atheroma plaques [8,9]. Treating these mice with a fusion protein comprising a type I TNF receptor, neutralizing the TNFα, also significantly reduces the size of atheroma plaques [9,10]. In humans, neutralizing TNFα using an anti-TNFα monoclonal antibody corrects endothelial dysfunction observed in chronic inflammatory diseases, such as RA and systemic vasculitis [11,12]. Furthermore, TNFα neutralization using either a fusion protein comprising a type II TNFα receptor or an anti-TNFα monoclonal antibody is associated with a reduction in the incidence of first cardiovascular events in RA patients [5].

Among the cellular agents of inflammatory response, mononuclear cells play an essential role in the development of atherosclerosis. Local inflammatory reaction within the atheroma plaque follows the phagocytosis by mononuclear cells of oxidized low density lipoproteins (LDLs) accumulated in the subendothelium [7]. This phagocytosis of oxidized LDLs is caused by scavenger receptors, in particular CD36 and scavenger receptor class A (SRA), and results in the formation of foam cells [13-15]. CD36 is strongly expressed by macrophages within the atheroma plaque [16]. The accumulation of oxidized LDLs by macrophages from subjects naturally deficient in CD36 appears clearly reduced [17]. Different cytokines essential for the regulation of inflammatory and immune responses modulate the expression of CD36 by macrophages. IL4 induces the expression of CD36 by activating the regulatory transcription factor peroxisome proliferator-activated receptor (PPAR)γ [18], while transforming growth factor beta represses it [19]. Redox signaling also plays a major role in regulating the expression of CD36. Various products derived from lipid peroxidation induce expression of CD36 by activating regulatory transcription factors, such as nuclear factor erythroid 2-related factor 2 (Nrf2), while vitamin E represses it [20-22]. Some therapeutic agents used in human pathology for their anti-inflammatory properties appear to modulate expression of CD36 by monocytes/macrophages and dendritic cells: aspirin induces expression of CD36 by human macrophages while dexamethasone induces expression of CD36 by dendritic cells [23,24].

These data highlight the key roles played by TNFα, mononuclear cells and scavenger receptors in the development of accelerated atherosclerosis observed in chronic human inflammatory diseases. New therapeutic agents that specifically neutralize TNFα have proved to be efficacious in the control of systemic inflammation and in reducing the incidence of cardiovascular events in RA patients [5]. These factors have prompted us to test the hypothesis that CD36 expression in human monocytes is regulated by TNFα and by adalimumab, a human anti-TNFα monoclonal antibody widely used in human pathology. Our work shows differential regulation of CD36 expression by TNFα and adalimumab. Characterizing the mechanisms involved in this differential regulation of CD36 expression may have implications for the prevention of high cardiovascular mortality observed in chronic inflammatory diseases.

## Materials and methods

### Isolation and culture of human monocytes

Peripheral blood mononuclear cells (PBMCs) were isolated from the cytapheresis residues obtained from healthy donors by density gradient on Lymphoprep (AbCys, Paris, France) according to the manufacturer's instructions. The monocytes were isolated from the PBMC by adhesion [25]. The PBMCs were cultured in macrophage-serum-free medium (M-SFM; Gibco Invitrogen, Cergy Pontoise, France) supplemented with L-glutamine at a concentration of 10^7 ^cells/ml for 1.5 hours at 37°C with 5% CO_2 _in a humid atmosphere. The non-adherent cells were eliminated via three PBS (Eurobio, Les Ulis, France) washes. The adherent cells (>85% of monocytes [26]) were then cultivated in M-SFM in 96-well trays (Becton Dickinson, Le Pont-De-Claix, France) with 0.125 × 10^6 ^monocytes/0.125 ml for reactive oxygen species (ROS) assay, in 24-well trays with 0.5 × 10^6 ^monocytes/0.5 ml for flow cytometry tests and gel shift assays, and in 12-well trays with 10^6 ^monocytes/ml for reverse transcription (RT)-PCR tests.

### Isolation of F(ab')2, the antigen binding fragment, from adalimumab

The isolation of the F(ab')2 fragment from adalimumab (Abbott France, Rungis, France), a human anti-TNFα IgG1 monoclonal antibody, was carried out by pepsin digestion using the ImmunoPure F(ab')2 Preparation Kit (Pierce by Interchim, Montluçon, France) according to the manufacturer's instructions. The purity of the F(ab')2 fragment obtained after adalimumab digestion was verified by migration of the final specimen on a 12% denaturant acrylamide gel, according to the manufacturer's instructions.

### Quantification of membrane expression of CD36 using flow cytometry

In time-course experiments, monocytes were incubated for 6 to 12 and 24 h with M-SFM alone or with M-SFM containing TNFα (10 ng/ml) or adalimumab (1 μg/ml). In additional assays, monocytes were incubated for 24 h with M-SFM alone or with M-SFM containing: human recombinant TNFα at increasing concentrations (0.1, 1 or 10 ng/ml; BD Biosciences Pharmingen, Le Pont-De-Claix, France); or a combination of TNFα (10 ng/ml) or adalimumab (1 μg/ml; a biologically relevant concentration used in human therapeutics [27]; Abbott France) with either TNFα (10 ng/ml), GW9662 (2 μM; Cayman Chemicals by Spi-Bio, Montigny le Bretonneux, France), Trolox^® ^(1 μM; Sigma-Aldrich, Saint Quentin Fallavier, France), or diphenylene iodonium chloride (DPI; 1 μM; Calbiochem by VWR International, Fontenay sous Bois, France). Rituximab, a human anti-CD20 IgG1 monoclonal antibody (Roche, Meylan, France) was used at the same concentration as adalimumab (1 μg/ml) as control antibody. To investigate the relative contributions of Fab and Fc fragments in the induction of CD36 membrane expression by adalimumab, monocytes were incubated for 24 h with the F(ab')2 fragment of adalimumab (0.8 μg/ml). Quantification of membrane expression of CD36 on monocytes was carried out using flow cytometry according to the following protocol: the monocytes were washed once with PBS and then incubated for 15 minutes at 4°C in 5 mM PBS EDTA and collected by aspirating and refilling the wells. The monocytes were then incubated with the anti-CD36 monoclonal antibody labeled with phycoerythrin (BD Bioscience Pharmingen), used at a ratio of 10 μl per 0.5 × 10^6 ^cells. The background staining was evaluated using a control isotype labeled with phycoerythrin (BD Bioscience Pharmingen) (data not shown). The region of interest of the monocyte population, comprising over 3,000 cells, was isolated on morphological criteria of cell size and granularity. The presence of strong CD14 expression, a characteristic of monocytes, was verified within the region of interest (data not shown). The quantification of membrane expression of CD36 was obtained from the geometric mean of the fluorescence measured [23].

### Study of PPARγ activation by gel-shift assay

Monocytes were incubated with M-SFM alone or with M-SFM containing TNFα (10 ng/ml) for 5, 30 or 60 minutes and then stimulated, or not, by a synthetic ligand of PPARγ, rosiglitazone (5 μM). Nuclear proteins were then isolated according to the following procedure: the monocytes were lysed at 4°C in a hypotonic buffer (10 mM Hepes Free Acid^® ^(Sigma-Aldrich), 10 mM KCl, 0.5 mM EDTA, 1 mM MgCl_2_, 0.1 mM EGTA) supplemented by anti-proteases (Complete^®^, Roche Diagnostics). Igepal^® ^(10%; Sigma-Aldrich) was added. The cytoplasmic extracts (supernatants) were isolated after centrifugation and the pellet was replaced in a hypertonic buffer, (20 mM Hepes Free Acid^®^, 400 mM NaCl, 0.5 mM EDTA, 1 mM MgCl_2_, 0.1 mM EGTA) supplemented with anti-proteases (Complete^®^) in order to extract the nuclear proteins. The proteins were dosed according to the Bradford method.

The oligonucleotide (Santa Cruz Biotechnology by Tebu-Bio, Le Perray en Yvelines, France) used for the shift had the following sequence: 5'-CAAAACTAGGTCAAAGGTCA-3', with the underlined sequence corresponding to the PPAR DNA consensus binding sequence. It was labeled with [γ-^32^P]ATP at 37°C using T4 polynucleotide kinase (Promega France, Charbonnières-les-Bains, France) and purified on appropriate columns (Quiagen, Courtaboeuf, France). The probe was labeled at 30,000 to 40,000 cpm/μl.

For the DNA protein reaction, 3 μg of proteins mixed at ambient temperature with the binding buffer (2 mM Hepes Free Acid^®^, 50 mM NaCl, 0.5 mM EDTA, 1 mM MgCl_2_, 4% glycerol, 2 μg/ml bovine serum albumin, 0.5 mM dithiothreitol), 3 μl of labeled oligonucleotides and 0.3 μg of poly (dI-dC) (Sigma-Aldrich) were added in a final volume of 25 μl and incubated for 20 minutes at room temperature. The specimens were placed on 5% non-denaturing acrylamide gel and set to migrate for 2.5 h at 180 V. The gel was dehydrated under vacuum and exposed by autoradiography.

### Analysis of CD36 mRNA expression using real time PCR

Monocytes were incubated for 4 h with M-SFM alone or with M-SFM containing either TNFα (10 ng/ml), adalimumab (1 μg/ml), or rosiglitazone (5 μM) with or without pretreatment with TNFα (10 ng/ml). The monocytes were lysed in TRIzol^® ^Reagent (Invitrogen) and the mRNA was extracted using the chloroform/isopropanol/ethanol standard procedure. To ascertain that RNA preparations were genomic DNA-free, a negative control reaction was systematically included in which the sample was substituted with water.

PCR for CD36 and β-actin cDNA was performed with the LC FastStart DNA master SYBR Green I (Roche Diagnostics). Amplification and detection were performed in a LightCycler^® ^system (Roche Diagnostics) as follows, according to the manufacturer's instructions. Reaction mixture (20 μl) was incubated initially for 8 minutes at 95°C to activate the Fast Start Taq DNA; amplifications were performed for 40 cycles (15 s at 95°C and 30 s at 69°C) for CD36 and β-actin. The primers were designed with the software Primer Express (Applied Biosystems, Foster City, USA). The primers were: 5'-TGT-AAC-CCA-GGA-CGC-TGA-GG-3' (sense) and 5'-GAA-GGT-TCG-AAG-ATG-GCA-CC-3' (antisense) for CD36; 5'-CCT-CAC-CCT-GAA-GTA-CCC-CA-3' (sense) and 5'-TGC-CAG-ATT-TTC-TCC-ATG-TCG-3' (antisense) for β-actin.

Real-time PCR data are represented as Ct values, where Ct is defined as the crossing threshold of PCR using Light-Cycler^® ^Data Analysis software. For calculating relative quantification of CD36 mRNA expression, we used the following procedure: ΔCtCD36 = CtSample - CtControl and ΔCtβ-actin = CtSample - CtControl; then, ΔΔCt represents the difference between ΔCtβ-actin and ΔCtCD36 calculated by the formula ΔΔCt = ΔCtβ-actin - ΔCtCD36; finally, the N-fold differential expression of CD36 mRNA samples compared to the control is expressed as 2^ΔΔCt^.

### Quantification of reactive oxygen species production

Monocytes were incubated for 1 h with Hanks balanced salt solution (HBSS; Eurobio, Les Ulis, France) alone or with HBSS containing adalimumab (1 μg/ml), TNFα (10 ng/ml) or F(ab')2 (0.8 ng/ml). ROS production was quantified by chemiluminescence in the presence of 5-amino-2,3-dihydro-1,4-phthalazinedione (66 mM; Luminol^®^, Sigma-Aldrich) using a thermostatically (37°C) controlled luminometer (Wallac 1420 Victor2, Finland) [26]. The generation of chemiluminescence was monitored continuously for 1 h. Results are expressed as total chemiluminescence emission (area under the curve).

### Statistical analysis

All flow cytometry and real time PCR experiments were performed at least three times. The values are expressed as the mean ± standard deviation (SD). A Wilcoxon test was used to assess the significance of differences between two conditions. All *p *values are two-sided, and *p *values less than 0.05 are considered significant.

## Results

### Regulation of CD36 membrane expression by TNFα and adalimumab

**Figure 1:**
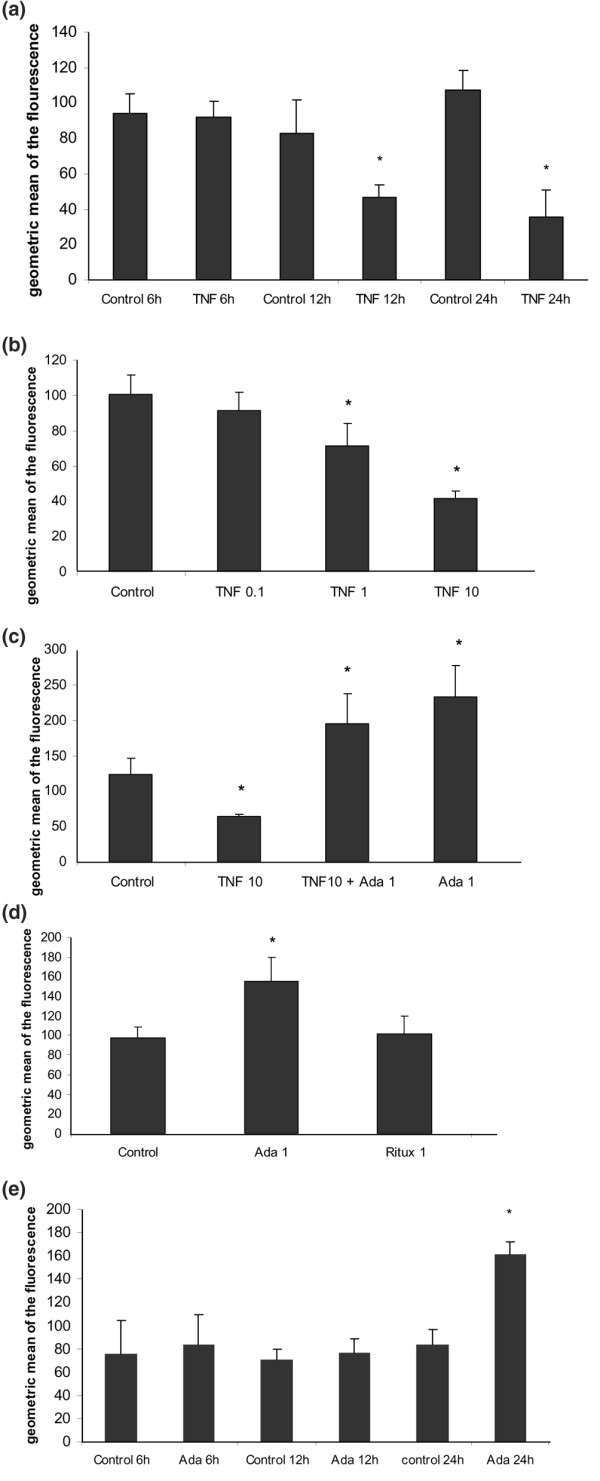
Regulation of CD36 membrane expression by tumor necrosis factor (TNF)α and adalimumab (Ada). **(a) **TNFα reduces the membrane expression of CD36: time course. Human monocytes were incubated with macrophage-serum-free medium (M-SFM) alone (control), or with M-SFM containing TNFα (10 ng/ml) for 6 to 12 and 24 h. Membrane expression of CD36 was quantified using flow cytometry. Data represent the geometric mean ± standard error (SE) of the fluorescence measured in three experiments in duplicate. *Significantly different from control (*p *< 0.05). **(b) **TNFα reduces membrane expression of CD36: dose effect. Human monocytes were incubated for 24 h with M-SFM alone (control), or with M-SFM containing TNFα at increasing concentrations (0.1, 1, or 10 ng/ml) for 24 h. Membrane expression of CD36 was quantified using flow cytometry. Data represent the geometric mean ± SE of the fluorescence measured in four experiments. *Significantly different from control (*p *< 0.05). **(c) **The reduction of membrane expression of CD36 induced by TNFα is inhibited by adalimumab independently of TNFα. Human monocytes were incubated with M-SFM alone (control), or with M-SFM containing either TNFα alone (10 ng/ml), TNFα combined with adalimumab (Ada; 1 μg/ml) or adalimumab alone for 24 h. Membrane expression of CD36 was quantified using flow cytometry. Data represent the geometric mean ± SE of the fluorescence measured in four experiments. *Significantly different from control (*p *< 0.05). **(d) **The increase in CD36 membrane expression induced by adalimumab is antibody-specific. Human monocytes were incubated with M-SFM alone (control), or with M-SFM containing either adalimumab (Ada; 1 μg/ml), or rituximab (Ritux; 1 μg/ml), a human anti-CD20 IgG1 monoclonal antibody, as a control antibody for 24 h. Membrane expression of CD36 was quantified using flow cytometry. Data represent the geometric mean ± SE of the fluorescence measured in four experiments in duplicate. *Significantly different from control (*p *< 0.05). **(e) **Adalimumab increases membrane expression of CD36: time course. Human monocytes were incubated with M-SFM alone (control), or with M-SFM containing adalimumab (Ada; 1 μg/ml) for 6 to 12 and 24 h. Membrane expression of CD36 was quantified using flow cytometry. Data represent the geometric mean ± SE of the fluorescence measured in three experiments in duplicate. *Significantly different from control (*p *< 0.05).

To study the effect of TNFα on the regulation of membrane expression of CD36, monocytes were treated with TNFα (10 ng/ml) for increasing periods of time and membrane expression of CD36 was quantified using flow cytometry. Figure 1a shows that TNFα did not influence membrane expression of CD36 after 6 h of cell culture in comparison to basal conditions (mean ± SD: 91.8 ± 9.2 versus 94.2 ± 11.2, -4%, *p *= 0.5). After 12 h, TNFα decreased membrane expression of CD36 (46.5 ± 6.9 versus 82.9 ± 18.5, -44%, *p *= 0.04). The strongest effect was observed after 24 h of cell culture, with a 67% TNFα-induced decrease of CD36 membrane expression (35.3 ± 15.5 versus 106.9 ± 11.3, *p *= 0.0004). Monocytes were then treated with TNFα at increasing concentrations for 24 h. Figure 1b shows that TNFα reduced membrane expression of CD36 in a dose-dependent manner: -9% (91.6 ± 10.3 versus 100.3 ± 10.9, *p *= 0.3), -29% (71.2 ± 13.1 versus 100.3 ± 10.9, *p *= 0.002) and -59% (41.4 ± 4.5 versus 100.3 ± 10.9, *p *= 0.003) for 0.1, 1 and 10 ng/ml TNFα, respectively, in comparison to basal conditions.

Next, we investigated whether the reduction of membrane expression of CD36 induced by TNFα could be inhibited by adalimumab. Figure 1c shows that adalimumab (1 μg/ml) inhibited the effect of TNFα (10 ng/ml) on membrane expression of CD36. Furthermore, adalimumab independently increased membrane expression of CD36 by 59% (194.9 ± 42.3 versus 122.8 ± 23.2, *p *= 0.03) in the presence of TNFα and by 90% (233.7 ± 49.7 versus 122.8 ± 23.2, *p *= 0.04) in the absence of TNFα, in comparison to basal conditions.

To assess the specificity of adalimumab's effect on membrane expression of CD36, we used rituximab, a human anti-CD20 IgG1 monoclonal antibody, as a control antibody. Figure 1d shows that adalimumab increased CD36 membrane expression (155.3 ± 24.1 versus 97.3 ± 11.7, +60%, *p *= 7 × 10^-5^) while rituximab did not influence it (101.3 ± 18.5 versus 97.3 ± 11.7, +4%, *p *= 0.48), in comparison to basal conditions. Figure 1e shows that adalimumab did not affect CD36 expression after 6 and 12 h, while it increased CD36 expression by 92% after 24 h of cell culture (161.5 ± 10 versus 84 ± 12.4, *p *= 0.0003) in comparison to basal conditions.

### Regulation of CD36 mRNA expression by TNFα and adalimumab

**Figure 2:**
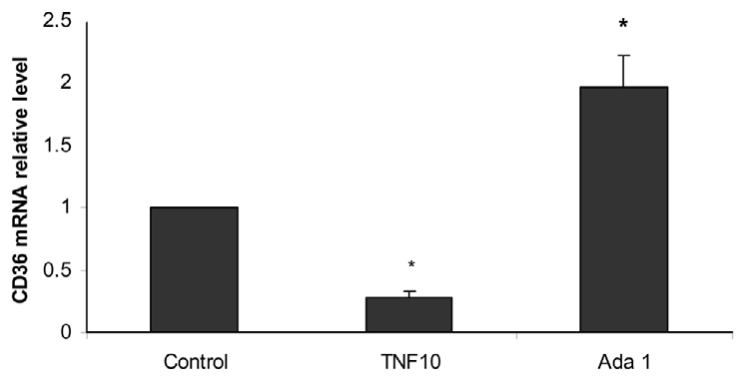
Regulation of CD36 mRNA expression by tumor necrosis factor (TNF)α and adalimumab. TNFα decreases CD36 mRNA expression and adalimumab increases CD36 mRNA expression. Human monocytes were incubated with macrophage-serum-free medium (M-SFM) alone (control), or with M-SFM containing TNFα (10 ng/ml) or adalimumab (Ada; 1 μg/ml) for 4 h. CD36 mRNA expression was quantified using RT-PCR and normalized to β-actin. Data represent the mean ± standard error of the relative quantification of CD36 mRNA expression measured in three experiments. *Significantly different from control (*p *< 0.05).

To study the effect of TNFα and adalimumab on CD36 mRNA expression, the monocytes were treated with TNFα or adalimumab and the relative quantification of CD36 mRNA was carried out by RT-PCR. Figure 2 shows that TNFα (10 ng/ml) reduced CD36 mRNA expression by 72% (mRNA relative level ± SD: 0.28 ± 0.05, *p *= 0.002), while adalimumab (1 μg/ml) increased CD36 mRNA expression by 96% (1.96 ± 0.2, *p *= 0.02) in comparison to basal conditions.

### Mechanisms involved in the regulation of CD36 expression by TNFα

**Figure 3:**
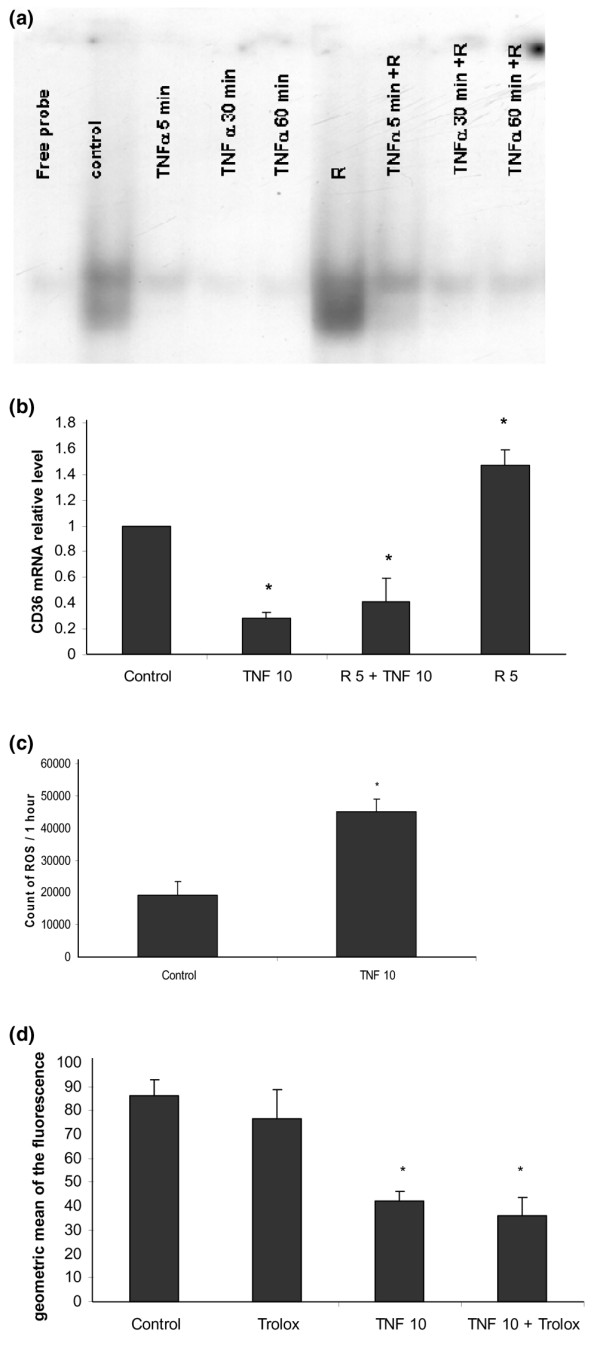
Mechanisms involved in the regulation of CD36 expression by tumor necrosis factor (TNF)α. **(a) **TNFα inhibits both basal and rosiglitazone-induced peroxisome proliferator-activated receptor (PPAR)γ activation. Human monocytes were incubated with macrophage-serum-free medium (M-SFM) alone (control), or with M-SFM containing TNFα (10 ng/ml) for 5, 30 or 60 minutes, and then stimulated, or not, with a synthetic ligand of PPARγ, rosiglitazone (R; 5 μmol/l) for 45 minutes. Nuclear proteins were isolated and a [γ-^32^P]ATP labeled oligonucleotide expressing the PPAR DNA consensus binding sequence was added. PPARγ activation was analyzed by gel-shift assay. **(b) **TNFα inhibits both basal and rosiglitazone-induced CD36 mRNA expression. Human monocytes were incubated with M-SFM alone (control), or with M-SFM containing TNFα (10 ng/ml) for 1 h and then stimulated or not with rosiglitazone (R; 5 μmol/l) for 4 h. CD36 mRNA expression was quantified using RT-PCR and normalized to β-actin. Data represent the mean ± standard error (SE) of the relative quantification of CD36 mRNA expression measured in three experiments. *Significantly different from control (*p *< 0.05). **(c) **TNFα induces reactive oxygen species production. Monocytes were incubated with Hanks balanced salt solution (HBSS) alone (control), or with HBSS containing TNF (10 ng/ml) for 1 h. Reactive oxygen species production was measured by chemiluminescence in the presence of 5-amino-2,3-dihydro-1,4-phthalazinedione in a thermostatically controlled luminometer. Data represent total chemiluminescence emission (area under the curve) for 1 h, measured in three experiments. *Significantly different from the control (*p *< 0.05). **(d) **The decrease in CD36 membrane expression induced by TNFα is not inhibited by an anti-oxidant (Trolox). Monocytes were incubated with M-SFM alone (control), or with M-SFM containing either TNFα (10 ng/ml), Trolox^® ^(1 μM), or TNFα combined with Trolox^® ^for 24 h and the membrane expression of CD36 expression was quantified using flow cytometry. Data represent the geometric mean ± SE of the fluorescence measured in three experiments in duplicate. *Significantly different from the control (*p *< 0.05).

Since PPARγ is a transcription factor that plays a key role in inducing membrane expression of CD36 on human monocytes [19], we investigated its implication in the regulation of CD36 expression by TNFα. We tested the hypothesis that PPARγ activation is inhibited by TNFα using gel-shift assays. The monocytes were incubated with M-SFM alone or with M-SFM containing TNFα (10 ng/ml) and stimulated, or not, with a synthetic ligand of PPARγ, rosiglitazone (5 μM), and PPARγ activation was analyzed by gel-shift assay. Figure 3a shows a basal activation of PPARγ that was inhibited by TNFα. Rosiglitazone increased the activation of PPARγ and this effect of rosiglitazone was inhibited by TNFα.

To evaluate the consequences of the inhibition of PPARγ activation by TNFα, we assessed the effect of TNFα on the induction of CD36 mRNA by rosiglitazone. Monocytes were incubated with M-SFM alone or with M-SFM containing TNFα (10 ng/ml) and stimulated, or not, with rosiglitazone (5 μM), and the relative quantification of CD36 mRNA expression was carried out by RT-PCR. Figure 3b shows that TNFα reduced CD36 mRNA expression (-72%, 0.28 ± 0.05, *p *= 0.002), while rosiglitazone increased CD36 mRNA expression (+46%, 1.46 ± 0.2, *p *= 0.02). The combination of TNFα and rosiglitazone inhibited the effect of the rosiglitazone by reducing CD36 mRNA expression (-59%, 0.41 ± 0.2, *p *= 0.002) in comparison to basal conditions.

Since redox signaling is involved in the regulation of CD36 expression in human monocytes [21,22], we analyzed its role in the repression of CD36 membrane expression induced by TNFα (Figure 3c,d). Monocytes were incubated with TNFα (10 ng/ml) and ROS production was quantified by chemiluminescence for 1 h. Figure 3c shows that TNFα induced a two-fold increase in ROS production in comparison to basal conditions (45,173 ± 3,966 versus 19,207 ± 4,115, *p *= 0.03). The role of ROS production in the regulation of CD36 expression induced by TNFα was analyzed using an antioxidant. Monocytes were treated with TNFα (10 ng/ml), or with an antioxidant derived from vitamin E, Trolox^® ^(1 μM), or with a combination of TNFα and Trolox^®^. Membrane expression of CD36 was then quantified using flow cytometry. Figure 3d shows that membrane expression of CD36 was not significantly modified by Trolox^® ^(76.6 ± 12 versus 86.2 ± 6.8, -12%, *p *= 0.11) in comparison to basal conditions. TNFα decreased CD36 expression (35.9 ± 7.8 versus 86.2 ± 6.8, -52%, *p *= 1 × 10^-5^) and this effect was not affected by Trolox^® ^(42. 3 ± 4 versus 86.2 ± 6.8, -60%, *p *= 6 × 10^-6^).

### Mechanisms involved in the regulation of CD36 expression by adalimumab

**Figure 4:**
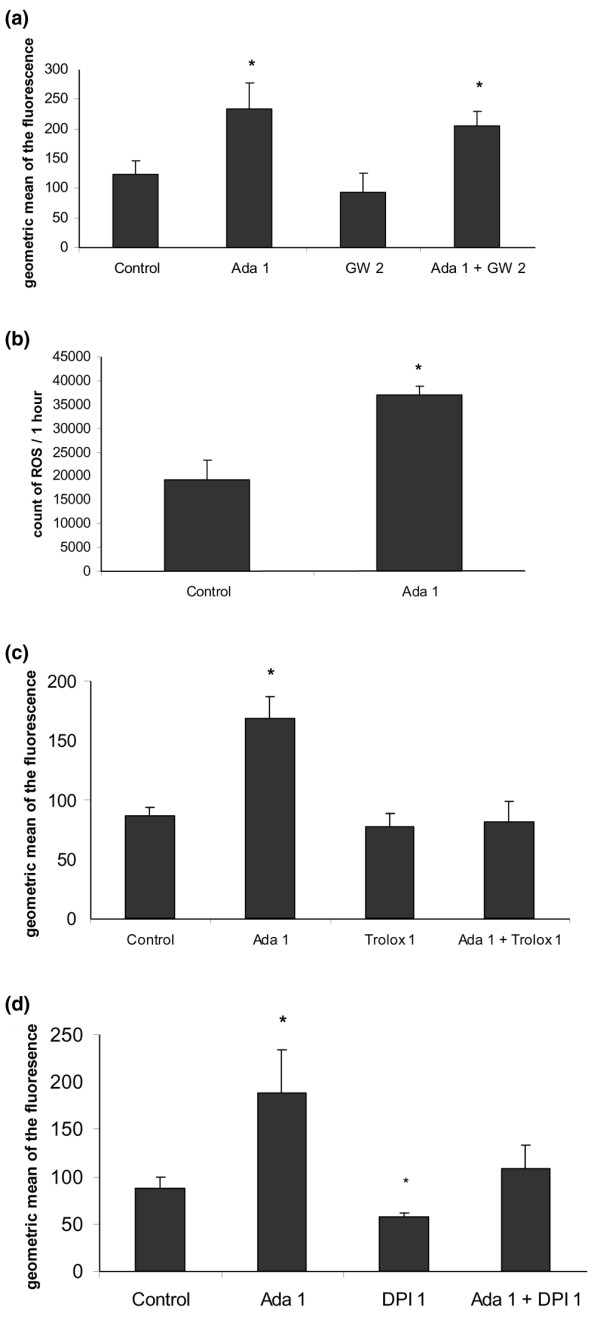
Mechanisms involved in the regulation of CD36 expression by adalimumab. **(a) **The increase in CD36 membrane expression induced by adalimumab is not inhibited by a peroxisome proliferator-activated receptor (PPAR)γ antagonist (GW9662). Monocytes were incubated with macrophage-serum-free medium (M-SFM) alone (control), or with M-SFM containing either adalimumab (Ada; 1 μg/ml), GW9662 (GW; 2 μM), or adalimumab combined with GW9662 for 24 h, and the membrane expression of CD36 was quantified using flow cytometry. Data represent the geometric mean ± standard error (SE) of the fluorescence measured in three experiments in duplicate. *Significantly different from the control (*p *< 0.05). **(b) **Adalimumab induces ROS production. Monocytes were incubated with Hanks balanced salt solution (HBSS) alone (control), or with HBSS containing adalimumab (Ada; 1 μg/ml) for 1 h. Reactive oxygen species production was measured by chemiluminescence in the presence of 5-amino-2,3-dihydro-1,4-phthalazinedione in a thermostatically controlled luminometer. Data represent total chemiluminescence emission (area under the curve) for 1 h, measured in three experiments. *Significantly different from the control (*p *< 0.05). **(c) **The increase in CD36 membrane expression induced by adalimumab is inhibited by an anti-oxidant (Trolox). Monocytes were incubated with M-SFM alone (control), or with M-SFM containing either adalimumab (Ada; 1 μg/ml), Trolox^® ^(1 μM), or adalimumab combined with Trolox^® ^for 24 h and the membrane expression of CD36 was quantified using flow cytometry. Data represent the geometric mean ± SE of the fluorescence measured in three experiments in duplicate. *Significantly different from the control (*p *< 0.05). **(d) **The increase in CD36 membrane expression induced by adalimumab is inhibited by a NADPH inhibitor (diphenylene iodonium chloride (DPI)). Monocytes were incubated with M-SFM alone (control), or with M-SFM containing either adalimumab (Ada; 1 μg/ml), DPI (1 μM), or adalimumab combined with DPI for 24 h and the membrane expression of CD36 was quantified using flow cytometry. Data represent the geometric mean ± SE of the fluorescence measured in three experiments in duplicate. *Significantly different from the control (*p *< 0.05).

Since PPARγ is a transcription factor that plays a key role in inducing membrane expression of CD36 on human monocytes [19], we investigated its implication in the induction of CD36 membrane expression by adalimumab (Figure 4a). The monocytes were treated with adalimumab (1 μg/ml) or with a PPARγ antagonist, GW9662 (2 μM), or with a combination of adalimumab and GW9662, and CD36 expression was quantified using flow cytometry. Figure 4a shows that adalimumab increased CD36 membrane expression (233.7 ± 43.7 versus 122.8 ± 23.2, +70%, *p *= 0.02), while GW9662 did not significantly decrease CD36 membrane expression (93.8 ± 31.8 versus 122.8 ± 23.2, -24%, *p *= 0.2), in comparison to basal conditions. The combination of adalimumab and GW9662 did not inhibit the effect of adalimumab on CD36 membrane expression, which remained increased (205.5 ± 24.3 versus 122.8 ± 23.2, +67%, *p *= 0.003). We assessed the effect of adalimumab on PPARγ activation and did not observe any effect in gel shift experiments (data not shown).

To evaluate the role of redox signaling in the induction of CD36 expression observed with anti-TNFα, monocytes were incubated with adalimumab (1 μg/ml) and ROS production was quantified by chemiluminescence for 1 h. Figure 4b shows that adalimumab induced a two-fold increase in ROS production in comparison to basal conditions (37,095 ± 1,693 versus 19,207 ± 4,115 *p *= 0.008). The role of redox signaling in CD36 expression was investigated by using an antioxidant. The monocytes were treated with adalimumab (1 μg/ml) or with an antioxidant derived from vitamin E, Trolox^® ^(1 μM), or with a combination of adalimumab and Trolox^®^, and membrane expression of CD36 was quantified using flow cytometry. Figure 4c shows that adalimumab increased CD36 membrane expression (168.6 ± 18.2 versus 86.23 ± 6.8, +96%, *p *= 0.001), while Trolox^® ^did not significantly modify it (76.7 ± 12 versus 86.23 ± 6.8, -11%, *p *= 0.06), in comparison to basal conditions. By contrast, the combination of adalimumab and Trolox^® ^inhibited the effect of adalimumab on CD36 membrane expression, which returned to levels observed in basal conditions (81.3 ± 17 versus 86.23 ± 6.8, -6%, *p *= 0.5).

Since NADPH oxidase is a key enzyme of oxidative metabolism, inducing production of free radicals in monocytes [28], we investigated its implication in the induction of CD36 membrane expression by adalimumab (Figure 4d). Monocytes were treated with adalimumab (1 μg/ml) or with an NADPH oxidase inhibitor, DPI, or with a combination of adalimumab and DPI, and the membrane expression of CD36 was quantified using flow cytometry. Figure 4d shows that adalimumab increased CD36 membrane expression (188.6 ± 46 versus 88 ± 10.9, +114%, *p *= 0.002), while DPI decreased it (56.9 ± 4 versus 88 ± 10.9, -35%, *p *= 0.0003), in comparison to basal conditions. In contrast, the combination of adalimumab and DPI inhibited the effect of adalimumab on CD36 membrane expression, which returned to levels observed in basal conditions (108.3 ± 25.4 versus 88 ± 10.9, +23%, *p *= 0.07).

**Figure 5:**
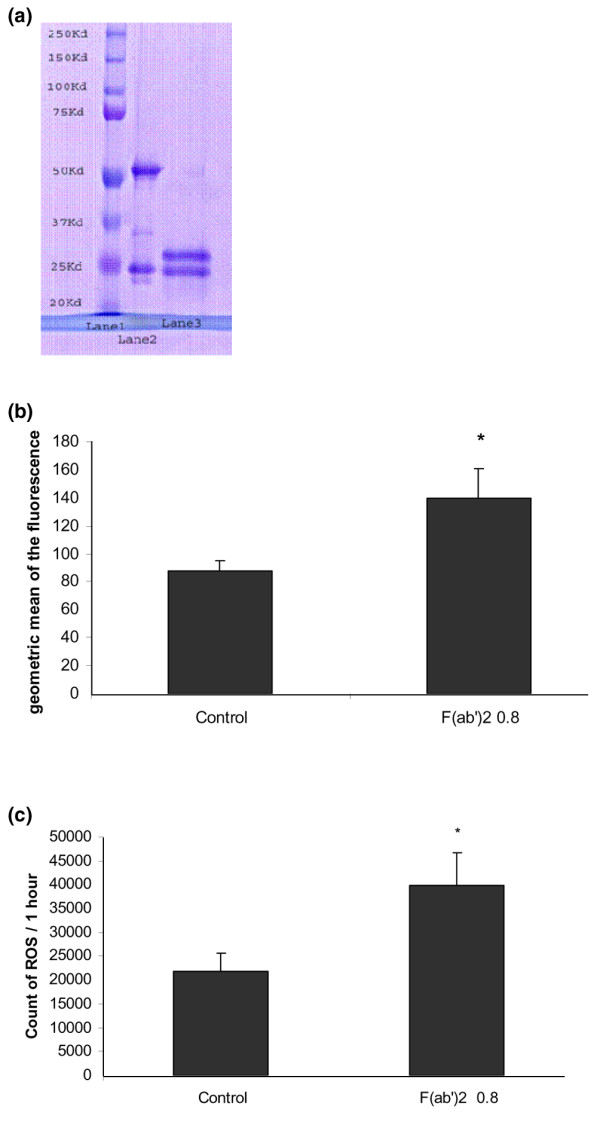
Role of the Fc portion of adalimumab in the regulation of CD36 expression. **(a) **Isolation of F(ab')2, the antigen binding fragment, from adalimumab with pepsin digestion. The purity of the F(ab')2 fragment obtained was verified by migration of the specimens obtained on a 12% denaturant acrylamide gel according to the manufacturer's instructions. Lane 1 represents the standard molecular weight (20 to 250 kDa). Lane 2 represents the light chain (25 kDa) and the heavy chain (50 kDa) of adalimumab. Lane 3 represents the intact light chain (25 kDa) and truncated heavy chain (30 kDa) obtained after pepsin digestion. **(b) **The F(ab')2 fragment of adalimumab increases membrane expression of CD36. Monocytes were incubated with macrophage-serum-free medium (M-SFM) alone (control), or with M-SFM containing the purified F(ab')2 fragment from adalimumab at an equimolar concentration to that of 1 μg/ml adalimumab (0.8 μg/ml of F(ab')2 fragment being equivalent to 1 μg/ml of adalimumab) for 24 h and membrane expression of CD36 in monocytes was quantified using flow cytometry. Data represent the geometric mean ± standard error of the fluorescence measured in three experiments in duplicate. *Significantly different from the control (*p *< 0.05). **(c) **The F(ab')2 fragment of adalimumab induces reactive oxygen species (ROS) production. Monocytes were incubated with Hanks balanced salt solution (HBSS) alone (control), or with HBSS containing F(ab')2 (0.8 μg/ml) for 1 h. ROS production was measured by chemiluminescence in the presence of 5-amino-2,3-dihydro-1,4-phthalazinedione in a thermostatically controlled luminometer. Data represent total chemiluminescence emission (area under the curve) for 1 h, measured in three experiments. *Significantly different from the control (*p *< 0.05).

The biological effects of monoclonal antibodies, such as adalimumab, involve both Fab and Fc fragments. The interaction between the Fc fragments of monoclonal antibodies and the Fc gamma receptor (FcγR) can activate redox signaling via NADPH oxidase [29,30]. To investigate the relative contributions of Fab and Fc fragments in the induction of CD36 membrane expression by adalimumab, we removed the Fc region from adalimumab through pepsin digestion and isolated the F(ab')2 region (Figure 5a). The monocytes were treated with the purified F(ab')2 fragment from adalimumab at an equimolar concentration to that of 1 μg/ml adalimumab (0.8 μg/ml of F(ab')2 fragment being equivalent to 1 μg/ml of adalimumab), and the membrane expression of CD36 was quantified using flow cytometry. Figure 5b shows that the F(ab')2 fragment of adalimumab increased membrane expression of CD36 (140 ± 20.7 versus 87.5 ± 7.4, +60%, *p *= 0.005) in comparison to basal conditions. Finally, we investigated whether the F(ab')2 fragment of adalimumab promoted ROS production. Monocytes were incubated with the F(ab')2 fragment of adalimumab (0.8 μg/ml) and ROS production was quantified by chemiluminescence for 1 h. Figure 5c shows that the F(ab'2) fragment induced a two-fold increase in ROS production in comparison to basal conditions (39,826 ± 6,927 versus 21,873 ± 3,834, *p *= 0.01).

## Discussion

Our work demonstrates differential regulation of CD36 expression by TNFα and adalimumab in human monocytes. TNFα inhibits both CD36 membrane expression and mRNA expression. The inhibition of CD36 expression by TNFα involves a reduction in PPARγ activation. Adalimumab independently increases both CD36 membrane expression and mRNA expression. The induction of CD36 expression involves redox signaling via NADPH oxidase activation.

Our study shows that TNFα inhibits both CD36 membrane expression and mRNA expression in human monocytes. Various studies have already shown modulation of CD36 by different cytokines. TNFα and IL1 reduce transcription of fatty acid translocase, homologous to CD36, in hamster adipocytes [31]. IL4 increases CD36 expression in murine macrophages [18] and transforming growth factor beta and IL10 reduce CD36 expression in human macrophages [32,33].

Our study suggests that the inhibition of CD36 expression by TNFα in human monocytes involves a reduction in PPARγ activation. A link between PPARγ and membrane expression of CD36 has already been established in murine macrophages, where deficiency in 12/15 lipoxygenase, an enzyme necessary to generate natural PPARγ ligands, led to a reduction in the expression of CD36 [18]. A reduction in PPARγ activation by TNFα has already been reported in human adipocytes and hepatocytes [34,35], but has not yet been documented in human monocytes. While IL4, a TH2 cytokine, induces CD36 expression via synthesis of natural PPARγ ligands in murine macrophages [18], we suggest that TNFα, a TH1 cytokine, inhibits CD36 expression via reduction of PPARγ activation in human monocytes.

Experimental data show that metabolites produced in an oxidative context increase the expression of CD36 in murine macrophages [21]. We demonstrate here that TNFα, which induces ROS production, decreases CD36 expression and that this effect is not altered by antioxidant. These results suggest that ROS production is not involved in the repression of CD36 induced by TNFα.

Our study shows that adalimumab increases both CD36 membrane expression and mRNA expression in human monocytes. This effect is antibody-specific while rituximab, an IgG1 human antibody directed against CD20, does not influence CD36 membrane expression. The effect of anti-TNFα antibodies on scavenger receptors had not been evaluated before now. Previous work reported that certain pharmacological agents, whose anti-inflammatory properties are used in human therapy, regulate *in vitro *CD36 expression. Aspirin increases CD36 expression in lines of human THP1 macrophages [23], dexamethasone induces CD36 expression in human dendritic cells from healthy subjects [24] and atorvastatin increases CD36 expression in human monocytes [36,37].

According to the results of our study, the increase in membrane expression of CD36 induced by adalimumab involves a redox signaling pathway via NADPH oxidase activation, but not PPARγ. This is in accordance with previous studies showing that products derived from lipid peroxidation induce transcription of CD36 in murine macrophages by activating transcription factors, such as Nrf2 [21,38]. On the other hand, the administration of antioxidants, such as vitamin E, leads to a reduction in CD36 expression in murine peritoneal macrophages, and human endothelial cells, and macrophages derived from human monocytes [20,22,39].

Although part of the biological effect of antibodies used in human therapy implicates their binding to FcγR [29,40], and binding of the Fc fragment to FcγR activates NADPH oxidase [30,41], the mechanism by which adalimumab increases membrane expression of CD36 appears independent of its Fc fragment. Indeed, the induction of CD36 by adalimumab was specific to the F(ab')2 portion. The F(ab')2 fragment increases membrane expression of CD36 and induces ROS production, suggesting that F(ab')2 and native antibody use the same signaling pathway. The slightly lower induction of CD36 expression observed with the F(ab')2 fragment in comparison to the native antibody could be explained by a partial alteration of the F(ab')2 fragments during the pepsin digestion process [42].

We suggest that the F(ab')2 effect on CD36 expression may partially be the consequence of binding of this fragment of adalimumab to transmembrane TNFα. This binding leads to the activation of various intracellular signaling pathways, in particular calcium-dependant pathways, and play a role in the biological activity of anti-TNFα monoclonal antibodies [43,44]. Such a reverse signaling phenomenon, resulting from the binding of adalimumab to transmembrane TNFα, could account for the differential regulation of CD36 expression by TNFα and adalimumab in human monocytes [45].

Differential regulation of CD36 expression by TNFα and adalimumab in human monocytes may have consequences on the high cardiovascular mortality observed in chronic inflammatory diseases such as RA. In such conditions, anti-TNFα agents appear to reduce the incidence of cardiovascular events [5]. In addition to their anti-inflammatory effect, which seems beneficial in atherosclerosis, anti-TNFα agents could correct endothelial dysfunction and lipid profile abnormalities reported in chronic inflammatory diseases [11,46]. The increase in CD36 expression induced by adalimumab reported in our study could contribute to the modulation of cardiovascular risk under anti-TNFα therapies. In murine models of atherosclerosis, ApoE-/- mice, the consequences of inactivating the gene encoding CD36 remain contradictory: a decrease in the formation of atheroma plaques in one case [47], and an increase in the size of atheroma plaques in another [48]. In humans, subjects naturally deficient in CD36 show greater atherosclerosis, which suggests CD36 has an anti-atherogenic role [49]. The increase in CD36 expression induced *in vitro *by pharmacological agents such as aspirin and atorvastatin, whose anti-atherogenic effects are clearly established in human therapy, would suggest that this may be the case [23,37].

## Conclusion

Our work demonstrates differential regulation of CD36 expression by TNFα and adalimumab in human monocytes. While TNFα inhibits both CD36 membrane expression and mRNA expression, an anti-TNFα monoclonal antibody, adalimumab, independently increases both CD36 membrane expression and mRNA expression. Better understanding of the impact of anti-inflammatory therapeutic agents, such as anti-TNFα, on scavenger receptors, such as CD36 and SRA, and membrane reverse cholesterol transporters, such as ATP-binding cassette transporters A1 (ABCA1), may have implications for the prevention of high cardiovascular mortality observed in chronic inflammatory diseases.

## Abbreviations

ABCA1 = ATP-binding cassette transporters A1; DPI = diphenylene iodonium chloride; FcγR = Fc gamma receptor; HBSS = Hanks balanced salt solution; IL = interleukin; LDLs = low density lipoproteins; M-SFM = macrophage-serum-free medium; Nrf2 = nuclear factor erythroid 2-related factor 2; PBMC = peripheral blood mononuclear cell; PBS = phosphate-buffered saline; PPAR = peroxisome proliferator-activated receptor; RA = rheumatoid arthritis; ROS = reactive oxygen species; RT-PCR = reverse transcription PCR; SD = standard deviation; SRA = scavenger receptor class A; TNF = tumor necrosis factor.

## Competing interests

The authors declare that they have no competing interests.

## Authors' contributions

JFB was involved in the design of the study, performed all the experiments and wrote the manuscript. PB was involved in performing cell cultures, flow cytometry and gel shift assays, and reviewed the article critically. HA was involved in performing RT-PCR. BF was involved in F(ab')2 isolation. JB isolated PBMCs from healthy donors. BM revised the article critically. JLD was involved in the design of the study and reviewed the article critically. AC was involved in the conception and design of this study, in the interpretation of data and reviewed this article critically. BP and ArC co-directed the conception and design of this study, participated in the interpretation of data and in the preparation of the manuscript and gave final approval of the manuscript for publication. All authors read and approved the final manuscript.
